# MEG Can Map Short and Long-Term Changes in Brain Activity following Deep Brain Stimulation for Chronic Pain

**DOI:** 10.1371/journal.pone.0037993

**Published:** 2012-06-04

**Authors:** Hamid R. Mohseni, Penny P. Smith, Christine E. Parsons, Katherine S. Young, Jonathan A. Hyam, Alan Stein, John F. Stein, Alexander L. Green, Tipu Z. Aziz, Morten L. Kringelbach

**Affiliations:** 1 University Department of Psychiatry, University of Oxford, Oxford, United Kingdom; 2 Institute of Biomedical Engineering, School of Engineering Science, University of Oxford, Oxford, United Kingdom; 3 Center of Functionally Integrative Neuroscience (CFIN), Aarhus University, Aarhus, Denmark; 4 Department of Neurosurgery, John Radcliffe Hospital, Oxford, United Kingdom; 5 Department of Psychiatry, Oxford Centre for Human Brain Activity (OHBA), University of Oxford, Oxford, United Kingdom; University College London, United Kingdom

## Abstract

Deep brain stimulation (DBS) has been shown to be clinically effective for some forms of treatment-resistant chronic pain, but the precise mechanisms of action are not well understood. Here, we present an analysis of magnetoencephalography (MEG) data from a patient with whole-body chronic pain, in order to investigate changes in neural activity induced by DBS for pain relief over both short- and long-term. This patient is one of the few cases treated using DBS of the anterior cingulate cortex (ACC). We demonstrate that a novel method, null-beamforming, can be used to localise accurately brain activity despite the artefacts caused by the presence of DBS electrodes and stimulus pulses. The accuracy of our source localisation was verified by correlating the predicted DBS electrode positions with their actual positions. Using this beamforming method, we examined changes in whole-brain activity comparing pain relief achieved with deep brain stimulation (DBS ON) and compared with pain experienced with no stimulation (DBS OFF). We found significant changes in activity in pain-related regions including the pre-supplementary motor area, brainstem (periaqueductal gray) and dissociable parts of caudal and rostral ACC. In particular, when the patient reported experiencing pain, there was increased activity in different regions of ACC compared to when he experienced pain relief. We were also able to demonstrate long-term functional brain changes as a result of continuous DBS over one year, leading to specific changes in the activity in dissociable regions of caudal and rostral ACC. These results broaden our understanding of the underlying mechanisms of DBS in the human brain.

## Introduction

The implantation of electrodes to stimulate areas deep in the brain has become the basis of highly successful therapies to alleviate the symptoms of otherwise treatment-resistant disorders such as chronic pain, Parkinson’s disease (PD), tremor and dystonia [1], [2]. Despite its remarkable clinical potential, the neural mechanisms underlying deep brain stimulation (DBS) are not well understood but translational research has shown that DBS directly changes brain activity in a controlled manner [3] and that, in principle, the resulting effects are reversible [4].

Neuroimaging techniques such as MEG are crucial to our understanding of the mechanisms underlying DBS, and in particular to identify potential surgical targets for electrode placement. It is a non-invasive technology, risk-free for DBS patients, and can provide novel information about the underlying whole-brain activity [2]. MEG uses superconducting quantum interference devices (SQUIDs) to measure the tiny magnetic components produced by neural activity [5]. MEG therefore provides a direct measure of electrical activity of the brain, and compared to other neuroimaging techniques, such as fMRI, has excellent temporal resolution on the scale of milliseconds. The main disadvantage of MEG, however, is its relatively poor spatial resolution. Advances in source analysis such as beamforming, however, have made it possible to localise brain activity with acceptable spatial resolution [6].

The severe artefacts related to the DBS device and its electrical activity have, until recently, precluded the use of MEG to investigate the effects of DBS. Litvak and colleagues have suggested that artefacts mainly originate from the percutaneous extension wire, made of stainless steel, used to connect the electrodes to the battery implanted in the abdominal region [7]. DBS electrodes made of titanium may cause less interference than those made of stainless steel with MEG recording.

To date, there has been paucity of studies investigating the effects of stimulation on brain activity using MEG. In 2006, our group carried out the first MEG study of a patient suffering from severe phantom limb pain [8]. This study used Synthetic Aperture Magnetometry (SAM), an adaptive beamforming technique, to localise neural activity when the stimulator was ON and OFF. We also used MEG to investigate brain activity during high-frequency DBS in a cluster headache patient [9]. Consistent with the study of the phantom limb pain patient, the data were analysed using the SAM method. Increased brainstem activity was reported in the periaqueductal gray (PAG) in the 10–20Hz frequency band when the patient was in pain as a result of the stimulator being turned off. In another study we also demonstrated correlation between the pain ratings reported by the patient and MEG signal power in the theta (6–9Hz) and beta bands (12–30Hz) [10]. Using MEG with movement disorder is also highly challenging. Makela and colleagues used signal space separation with temporal extension (tSSS) to denoise highly noise-contaminated MEG data, and subsequently studied the effects of DBS in a PD patient [11]. Dynamic imaging of coherent sources (DICS) beamforming has also been employed recently for MEG recordings taken simultaneously with local field potentials (LFPs) [12]. The data acquired from a PD patient were analysed to reconstruct the MEG sources and to estimate their coherence with the LFP signals. The results showed strong coherence in different frequency ranges. Similar results were obtained by Hirschmann, Schnitzler and colleagues who recorded LFPs during MEG and analysed with a frequency domain beamformer [7].

These studies have all reported that the severe artefacts induced by DBS devices impede accurate MEG analysis. Here, we describe a novel approach to the analysis of MEG recordings from a DBS patient during ON and OFF stimulation. Null beamforming allows us to localise sources of activity more precisely than with conventional beamforming approaches. The null-beamformer, also known as a sidelobe canceller, is a widely-used method in radar array processing [13]. It has been shown to be effective at localising coherent MEG sources that are poorly reconstructed using conventional beamforming [14], has been further validated in MEG source localisation with auditory data [15]. Here, however, we use it in a different context: to suppress spatially the noise that is generally distributed near the burr holes where the DBS electrodes are implanted. We first test the accuracy of the null-beamforming by predicting the location of the implanted DBS electrodes with this method, and comparing this to the actual location, as identified with anatomical imaging. To the best of our knowledge, this is the first time that electrodes implanted in the brain at known locations have been used to test the accuracy of a source localisation method.

This novel analysis method has allowed us to examine two sets of MEG recordings acquired one week and one year after surgery in a patient suffering from whole-body chronic pain. The patient is one of the first cases in which DBS electrodes have been implanted in the anterior cingulate cortex (ACC). Understanding the brain changes caused by stimulation of this area represents a significant advance to our understanding of the mechanisms of action underlying DBS. We directly compared the results of source localisation of MEG during stimulation (ON) and with stimulation turned off (OFF). In addition, we are able to directly compare the effects of DBS over the short and long term on activity in the network of brain regions implicated in the experience of pain. Longitudinal studies of this kind are also important in our understanding of DBS mechanisms over time.

## Results

### Testing the Accuracy of the Null-beamformer Method

DBS provides a unique opportunity to validate a source reconstruction method using the deep electrode implanted in the human brain. This can be achieved by localising the MEG sources at the fundamental frequency of the applied stimulation, in this case at 130Hz, using beamforming methods (see *Materials and methods*). The sources should then be at the same locations as the electrode identified using the co-registration of CT and MR images. This approach is useful in reconstructing sources deep in the brain.

After data were pre-processed and filtered in the range of 1-40Hz, the brain was divided into grid points and the power of each grid point was calculated using the LCMV beamformer (see Materials and methods). The whole 10-mintes recordings were used to estimate spectral spatial matrix. The location of the grid point with the maximum power was then considered as the location of the null. This matched the location of burr holes that obtained using MRI and CT images. The same location (i.e., lead-field) was used for the null during source reconstruction using the null-beamformer in all frequency bins (5, 10, 15 and 20Hz).

Our experiment showed that only one null is sufficient to cancel out the DBS artefact in this subject. However, in other experiments we may need to cancel out a set of grids points that are related to the artefacts. One strategy in this case would be identifying the first location as above and then identify the location of the second null by inspecting the power spectrum of the null-beamformer which uses the location of the first null. This procedure can be continued iteratively to identify the location of the remaining nulls. Another strategy would be to identify a region that corresponds to the artefact and then use the eigenvectors corresponding to the biggest eigenvalues of the lead-fields within that region as the lead-field of the nulls.

**Figure 1:**
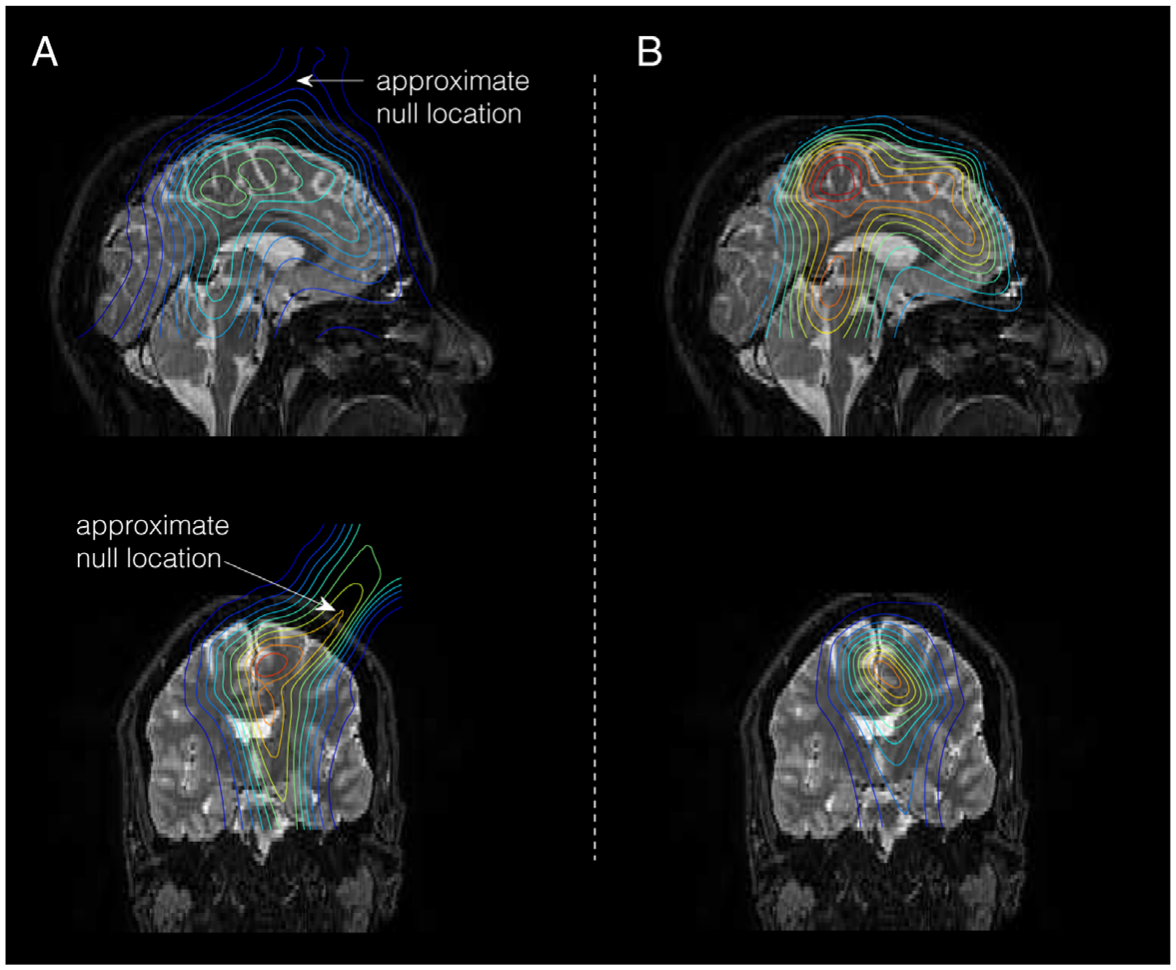
Application of the null-beamformer. The figure shows the estimated power of the sources in the mid-sagittal (top) and mid-coronal (bottom) view following the use of A) conventional beamformer and B) null-beamformer. The threshold value is 70% of the peak of the power spectrum. As can be seen, the null-beamformer has successfully removed the interference outside of the brain. Please note that in order to best depict the sources of brain activity, the null location is approximate and its actual location is in other anatomical planes (not shown).

Equation (4) from the section on *Materials and methods* was used to estimate the three-dimensional power spectrum as a measure of neural activity. The results were then thresholded and superimposed on the patient’s anatomical MRI. Figure 1 presents a comparison between results obtained using conventional beamforming (Figure 1A) and null-beamforming (Figure 1B).

**Figure 2:**
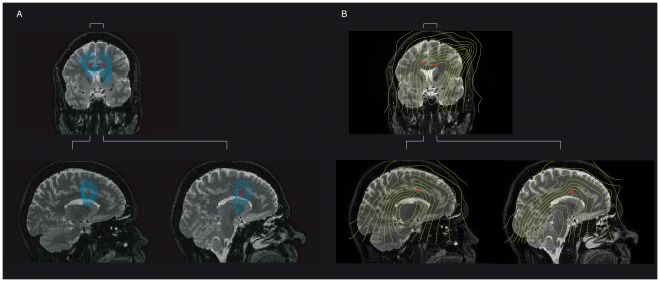
Comparison of the accuracy of using null and conventional beamformers for the localization of known locations of DBS electrodes. A) We used the null beamformer to localize the DBS electrodes when the stimulator was ON at 130 Hz. The coronal view of the lowest electrodes as localised on the patient’s MRI (red markers) compared with the overlay of contours of the estimated power using the null-beamformer. Two sagittal slices through lower left electrode and sagittal view of the lower right electrode. The fit is especially good on the left side. B) Similar localization using the conventional beamformer method shows a less good fit. In particular the method is unable to localize both electrodes.

The accuracy of our source localisation is illustrated in Figure 2 which compares the result of using the null-beamformer with a conventional beamformer. The actual locations of the lower electrodes following our registration procedure are shown by red markers and the reconstructed source powers are shown by contour lines. Figure 2A shows the result of using a null-beamformer with a coronal view of both electrodes and sagittal slices through the lower left electrode and a sagittal slice through the lower right electrode. Figure 2B shows similar slices when using a conventional beamformer. These examples broadly support our approach: rejecting the channels with large variations and then localising the sources using null-beamforming can suppress artefacts and accurately localise the known sources. Localisation of the right electrode was sligthly inaccurate for the null beamformer (see Figure 2A), which may be the result of head deviation and slight movement during the recording session. Overall, these observations support the efficacy of the method in spatially filtering out interference from the DBS device.

### Changes in Whole-brain Activity for Chronic Pain with and without DBS Stimulation

**Figure 3:**
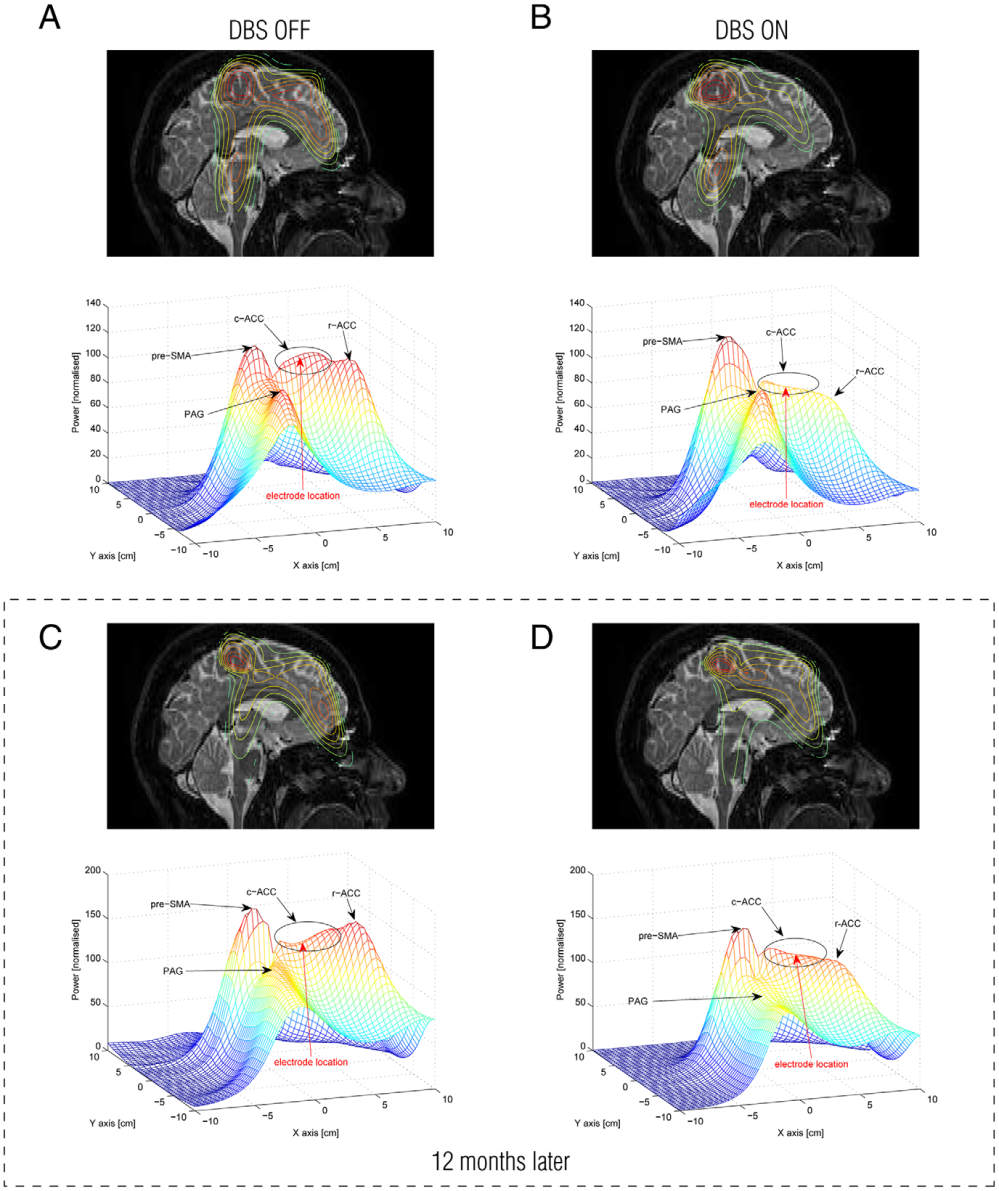
Summary of changes in brain activity between DBS ON and OFF after one week and one year after surgery. Four main midline brain regions were identified that had increases in power following pain related changes when DBS was OFF: the pre-supplementary motor area (pre-SMA), caudal-ACC (c-ACC), rostral-ACC (r-ACC) and brainstem (periaqueductal grey, PAG). A) The figure shows the mid-sagittal slice with the contours of the reconstructed MEG sources at 10Hz using null-beamformer with DBS OFF. Below each brain slice is shown the corresponding 3D mesh plot of the reconstructed neural activity in the mid-sagittal slice at 10Hz (with the lower electrode location inserted in red). B) Similarly, when DBS is ON, the figure shows the reconstructed MEG sources and the corresponding 3D mesh plot. This shows a decrease in activity in the caudal and rostral ACC with pain relief. C) One year later, after a continuous DBS, a similar pattern of changes in activity emerges when the patient is in pain (DBS OFF). D) Similarly, a decrease in activity in rostral ACC is evident following pain relief with DBS ON. Interestingly, the activity in the caudal ACC appears to be depressed during pain after one year of DBS and thus show a much smaller decrease in activity upon pain relief. This could be suggestive of plastic changes following long term DBS. Please note that the vertical axes (depicting normalised power) are the same for Figures A and B but not for Figures C and D. As can be seen from the figure, the power of the reconstructed sources is independent from the number of rejected channels (see Methods). It is also notable that the full width at half maximum (FWHM) of each peak is also independent from the number of rejected channels (e.g. see the pre-SMA). The number of rejected channels is therefore likely only to have a minor influence on the source space results.

We used MEG to investigate the changes in brain activity with DBS on and off, both short term (after one week) and long term (after one year). Figure 3 summarises the changes in brain activity after one week and one year in the mid-sagittal views. Figure 3A shows the reconstructed MEG sources at 10Hz using the null-beamformer when the DBS stimulator was turned OFF one week after surgery. This figure shows increased activity in the pain-related areas: pre-supplementary motor area (pre-SMA), brainstem (PAG) and medial prefrontal areas (mainly ACC) compared with stimulation turned on (Figure 3B). During stimulation at this time, the patient reported almost complete pain relief; concurrently, activity in the r-ACC in particular, and also in the c-ACC is substantially lower than during stimulation.

MEG recordings from the patient one year after surgery in the OFF condition are presented in Figure 3C. The electrode location can be identified from the contour plots in the c-ACC area. The posterior part of c-ACC as well as r-ACC show increased activity compared with that seen in the previous year’s MEG recordings. Analysis from the results of the ON condition are shown in Figure 3D. The main impact of the DBS is on activity in the r-ACC, which again is suppressed when the stimulator is turned ON. Brainstem activity also can be seen which has smaller, more focused activity compared to other sources.

Figure 3 also presents changes in brain activity using 3D mesh plots of the activity in mid-sagittal plane one week and one year after surgery. This figure shows that when the stimulator is turned ON one week after surgery, activity in both c-ACC and r-ACC (but not more posterior parts of c-ACC) significantly decreases. Interestingly, after one year of continuous DBS, the power of activity in c-ACC does not increase when DBS is OFF, contrasting with the results from one week post-surgery (see Figure 3A and 3C).

### Long-term Effects of DBS

In order to further characterise the effects of long-term stimulation, we calculated the mean amplitude of sources (and their variances) and source powers in different range of frequencies in the ON and OFF stimulation conditions across the short (one week, see Table 1A) and long term (one year, see Table 1B). We used a series of ANOVAs to investigate the effect of stimulation (ON and OFF), frequency (5Hz, 10Hz, 15hz and 20Hz), and time (one week and one year after surgery) and whether there were differences dependent on the region of the brain examined (location: Pre-SMA, c-ACC, r-ACC and PAG) on activity.

**Table 1:** Changes in activity in brain regions one week and one year after surgery.

A. One week after surgery									
				5Hz		10Hz	15Hz	20Hz	
Pre-SMA		DBS OFF		20.60 (12.18)		15.20 (10.72)	8.97 (3.32)	5.17 (0.80)	
		DBS ON		17.86 (7.49)		15.33 (9.11)	10.86 (5.87)	6.54 (1.68)	
c-ACC		DBS OFF		20.75 (16.4)		14.32 (8.15)	7.95 (2.13)	3.99 (0.41)	
		DBS ON		13.94 (3.11)		9.6 (1.64)	5.6 (0.46)	3.43 (0.11)	
r-ACC		DBS OFF		14.99 (9.39)		10.35 (3.57)	5.30 (1.13)	2.82 (0.22)	
		DBS ON		10.69 (4.49)		7.89 (1.79)	4.11 (0.63)	2.85 (0.07)	
PAG		DBS OFF		4.29 (8.34)		10.56 (2.81)	6.42 (0.80)	3.08 (0.2)	
		DBS ON		12.68 (2.07)		10.53 (1.46)	7.1 (0.79)	4.33 (0.30)	
**B. One year after surgery**									
			**5Hz**		**10Hz**		**15Hz**	**20Hz**	
Pre-SMA	DBS OFF		22.14 (3.9)		20.76 (4.05)		15.22 (1.7)	9.55 (0.52)	
	DBS ON		19.08 (5.4)		17.23 (6.63)		12.61 (4.07)	7.9 (1.847)	
c-ACC	DBS OFF		23.50 (3.13)		16.42 (1.23)		9.33 (0.44)	5.76 (0.21)	
	DBS ON		20.96 (3.57)		15.09 (0.80)		8.6 (0.27)	5.77 (0.64)	
r-ACC	DBS OFF		26.95 (8.76)		17.56 (2.27)		7.8 (0.32)	4.48 (0.13)	
	DBS ON		20.16 (9.48)		13.67 (4.12)		6.55 (0.28)	4.38 (0.17)	
PAG	DBS OFF		16.17 (9.42)		13.18 (3.1)		9.14 (0.66)	5.37 (0.12)	
	DBS ON		12.96 (3.39)		11.10 (2.42)		7.58 (1.26)	4.44 (0.42)	

The table shows the mean amplitudes (and their variances) and source powers in different range of frequencies in DBS ON and OFF conditions (A) one week after surgery and (B) one year after surgery.

Please note that in all reports of analyses of variance (ANOVA), the analyses incorporate Geisser-Greenhouse correction for inhomogeneity of variance [16], and that *F* ratios are reported with corrected degrees of freedom.

Applying a three-way ANOVA on the results of one week after surgery (Table 1A) showed a significant effect of stimulation [F(1,29) = 10.03, *p<0.001*] along with a significant interaction between location x frequency x stimulation [F(3.94,114.36) = 11.96, *p<0.05*]. A subsidiary ANOVA at 5 Hz showed also a significant effect of location [F(2.16,62.58) = 197.16, *p<0.001*] and an interaction between location and stimulation [F(2.05,59.42) = 23.28, *p<0.001*]. These effects arose because the differences between the ON and OFF stimulation were greater in c-ACC than in PAG or SMA, especially in the low frequencies. The results of ANOVA contrasting ON and OFF stimulations in c-ACC at 5Hz was F(1,29) = 60.05, *p<0.001*, indicating that the decrease of activity in this region is statistically significant in response to the stimulation.

Analysis of results of one year after surgery (see Table 1B) showed a significant effect of stimulation [F(1,17) = 36.79, *p<0.001*] and a significant interaction between location x frequency x stimulation [F(2.74,46.54) = 19.70, *p<0.001*]. At 5Hz, there was a significant effect of location [F(2.42,41.21) = 133.85, *p<0.001*] and a significant interaction between location and stimulation [F(2.68,45.49) = 13.19, *p<0.001*]. In addition to these effects, an ANOVA contrasting ON and OFF stimulations at 5Hz in the r-ACC revealed that the changes of the activity at this location is significant [F(1,17) = 44.22, *p<0.001*]. These analyses confirm the differences between the ON and OFF stimulation were greater in r-ACC than other locations. An ANOVA contrasting ON and OFF stimulation also showed a significant decrease of activity in c-ACC with F(1,17) = 15.37, *p<0.005*. There were no significant differences, however, in pre-SMA and PAG for any frequency.

We then compared the results from one week and one year after surgery using an ANOVA which gave rise a significant effect of location [F(2.42,41.167) = 482.29, *p<0.001*] and a significant interaction [F(3.03,51.46) = 17.46, *p<0.001*]. Overall, activity in r-ACC was greater one year after surgery compared to the first week after surgery [F(1,17) = 228.42, *p<0.001*]. Furthermore, there was a significant difference between the activity in the c-ACC over time [F(1,17) = 486.98, *p<0.001*].

## Discussion

Significant progress in understanding the principles of human brain function requires not only powerful correlative spatiotemporal neuroimaging methods but also causal methods for directly changing brain function. Deep brain stimulation (DBS) is one causal method which may help, especially if combined with a powerful functional neuroimaging technique such as MEG. In this paper we have demonstrated a novel method for overcoming technical obstacles related to combining DBS with MEG recording. This method has allowed us to map the functional changes in whole-brain activity linked to switching DBS ON and OFF. In addition, we have also been able to map the changes in whole-brain activity as a result of long-term use of DBS.

Here, we use a novel method, null-beamformer to deal with the artefacts related to the DBS electrodes when using MEG to examine changes in brain activity caused by DBS. We demonstrate that this method can successfully suppress artefacts to facilitate accurate localisation of electrode location in the human brain. This is possible because the signal of interest and noise have different, known spatial locations. In addition, the electrodes inside the brain appear to have no impact on the recorded MEG data. These key factors mean that the null-beamformer is both feasible and accurate. We also note that the majority of the channels rejected because of noise were around the burr holes and that data from these regions of the brain surface are missing. Therefore, MEG analysis in the source domain is likely to be preferable to that in the sensor domain.

We applied this novel analysis method to examining the differences in whole-brain activity in a chronic pain patient when DBS is ON (and providing pain relief) compared to when DBS is OFF (and the pain has returned). We found that the pain condition (DBS OFF) gave rise to changes in activity in a pain network comprising caudal and rostral ACC, the pre-supplementary motor area (pre-SMA), and the periaqueductal grey in the brainstem (PAG). In particular, we found that subsequent pain relief (DBS ON) was associated with statistically significant decrease in low frequency activity in both caudal and rostral ACC.

This pattern of activity fits well with the existing studies of the functional neuroanatomy of pain experience and relief. Human neuroimaging studies have shown that at least two dissociable parts of the ACC (r-ACC and c-ACC), appear to be involved in pain relief [17]. Speifically, activity in these two regions has been shown to increase during pain relief from thalamic DBS, studied using PET [18]. Our findings of PAG and the pre-SMA involvement are also consistent with fMRI studies of acute pain in healthy adults [19]. Further, in a large case-series we have demonstrated that implanting an electrode in the PAG can help some forms of treatment-resistent pain [20].

We were also able to compare the effects of stimulation one week and one year after surgery. We found that activity in rostral ACC was changed one year after surgery compared to the first week after surgery. This is interesting, given that the rostral ACC is understood to modulate the cognitive effects of pain [21] and has been implicated in the placebo effect for pain [22].

Activity in the caudal ACC appears to be depressed during the experience of pain after one year of DBS. The decrease in activity seen during pain relief (DBS ON) was much smaller at one year compared with one week. This could be suggestive of plastic brain changes following long term DBS and merits further investigation. The results are supportive of the hypothesis that DBS works primarily through rebalancing the resting state networks of the human brain [23], [24].

It should be noted that one important limitation of the present experiment is the different number of rejected channels which could result in unexpected and biased source activity from a number of potential sources. Such problems can for example arise from having a different location of a brain region in relation to the positions of the sensors because participants have different head shapes and thus position themselves differently in the scanner. Such variations may result in different lead-fields for the same anatomical location and therefore different weight or estimated power of the beamformer.

We have tried to minimize the impact of these potential confounds and are confident that the data has sufficient signal-to-noise-ratio. This conclusion is strengthened by consulting the four meshes plotted in Figure 3, from which it is clear that the power of the reconstructed sources is independent from the number of rejected channels. It is also notable that the full width at half maximum (FWHM) of each peak is independent from the number of rejected channels (e.g., see the SMA). Finally, it should be noted that we have not used beamforming weights to reconstruct the power of sources. We used output power of the LCMV beamformer and the normalised power of null-beamformer as a measure of neural activity.

## Conclusions

Our results provide several novel insights. First, we show that the null-beamformer can suppress noise that is usually spatially distributed around the burr holes from the DBS operation. Second, we identify a network of brain regions on the midline including the pre-SMA, PAG, caudal and rostral ACC as the dominant sources of activity during pain (when the stimulator was turned OFF). The activity in caudal and rostral ACC was subsequently suppressed in frequencies up to approximately 20Hz, when the patient experienced pain relief during stimulation. Third, these results confirm the importance role of dissociable regions of the ACC in the affective aspect of pain experience. Fourth, a comparison of the MEG data acquired one week and one year after surgery showed that activity in the caudal ACC is not suppressed to the same extent one year on, suggesting long-term brain changes after 12 months of continuous stimulation. Finally, the patient reported almost complete pain relief during the stimulation and concurrent neural activity as measured by the MEG was consistent with this self-report. Selected regions of the ACC, therefore, may be considered as suitable targets for DBS in the treatment of severe refractory chronic pain such as whole-body pain. Overall, MEG appears to be a safe non-invasive neuroimaging technique with excellent temporal resolution with the potential to advance our understanding mechanisms of DBS, in addition to revealing optimal surgical targets.

## Materials and Methods

### Ethics Statement

Ethical approval of the research methods was obtained on 18 July 2008 from Oxfordshire Research Ethics Committee A (Reference: 08/H0604/58), specifically covering this study. Participation was voluntary and the patient gave written informed consent for participation in the MEG recordings.

### Patient and Data Acquisition

MEG recordings were obtained from a a chronic pain patient who had undergone deep brain stimulation (DBS) surgery in 2009 at the John Radcliffe Hospital, Oxford, UK. The patient was a 50-years old male, who underwent DBS surgery after struggling with chronic whole-body pain for over 10 years as a result of high cervical injury following a motorbike accident.

The MEG recordings lasted approximately 25 minutes. Bilateral DBS in the caudal ACC–also known as posterior rostral cingulate zone (RCZp) [25] - applied at 5.0V in amplitude, pulse width of 300ms and a frequency of 130Hz gave the patient almost complete pain relief. This choice of high-frequency stimulation is a consequence of the site of electrode implantation. For DBS of regions such as periaqueductal grey (PAG) or sensory thalamus, it is possible to relieve phantom limb pain using a stimulation frequency of 20–40 Hz [20]. While the majority of movement disorder DBS patients receive high-frequency stimulation (130–180Hz) [26], using such high frequency stimulation in brainstem regions such as the PAG may lead to a significant worsening of the chronic pain.

### Surgical Procedures

Before surgery, we obtained anatomical MRIs, with a high-resolution T1 and T2 volumes with 1x1x1 mm voxel dimensions to plan the electrode implant protocol. The Cosman–Roberts–Wells stereotactic frame was applied to the patient’s skull under local anaesthetic. A stereotactic CT scan was then performed and the pre-operative MRI was combined with the stereotactic CT. The anterior and posterior commissures were identified on the axial images for the location of electrodes. The electrodes were model 3387 (Medtronic Neurological Division, Minneapolis, MN) with four platinum-iridium cylindrical surfaces (1.27mm diameter and 2.0mm exposed tip and 1.5mm gap between two adjacent electrode contacts). After placing the electrodes in the t target location, the whole stimulation system was connected using extension leads to the existing implanted pulse generator (IPG – Kinetra TM, Medtronic) which remained externalised for one week. Immediately after the operation, the patient had another CT scan to confirm that the electrodes had been implanted in their target locations. To determine the precise electrode location, the MRI and CT images were co-registered using fMRIB software library (FSL) using anatomical landmarks [27].

### MEG Recordings

The MEG recordings were collected using a 306-channel Elekta Neuromag system (204 planar gradiometers, 102 magnetometers) at the University of Oxford. Only data from the 102 magnetometers were analysed. This is because multimodal data sets have incommensurate measurement units and suffer from different levels of noise. This means that combining two sensor types without any correction does not necessarily improve the spatial resolution of reconstructed sources [28], [29]. An advantage of the magnetometer over the gradiometer is the sensitivity to the deep sources as a result of its sensor configuration.

Data were sampled at 1KHz with an anti-aliasing cut-off filter of 200Hz. Before recording, a three-dimensional digitizer (Polhemus Fastrack) was used to record the patient’s head shape relative to the position of the headcoils, with respect to three anatomical landmarks which could be registered on the MRI scan (the nasion, and the left and right preauricular points). No significant head movements were noted during scanning.

The patient was relaxed and seated comfortably during the recording. The patient’s stimulator was turned off for 30 minutes prior to scanning. For the first 10-minute scan, the patient’s stimulator was ‘on’ and set to 130Hz. After five minutes break with no recording, we recorded for a further 10 minutes with the stimulator turned ‘off’. MEG data were thus acquired in ten minute blocks of ‘on’ and ‘off’ stimulation. The patient reported that he was pain-free while the stimulator was ‘on’, but he reported severe pain in the ‘off’ condition. The MEG recordings took place one week after the initial DBS operation and again one year later.

### Pre-processing and Source Reconstruction

MEG data were pre-processed using SPM 8, Fieldtrip and Elekta Neuromag software. Data was visually inspected and saturated channels or channels with large variations were excluded. Twelve channels from the ON condition and 8 channels from the OFF condition were rejected from the data acquired one week after surgery, and 20 channels from the ON condition and 6 channels from the OFF condition were rejected from the data acquired one year after surgery, Theoretically, rejecting a small number of channels should have only a minimal impact on the source localisation with beamforming.

Head movement was kept to a minimum during recording, and head position was localised immediately before the start of the experiment. The sampling rate was 1 kHz, and data were filtered using a band-pass filter in the range of 1–40Hz. Independent component analysis (ICA) was used to remove eye blinks and movements. The ICA component related to eye blinks and movements were identified from their topographic maps and electrooculogram channel. Furthermore, the data set were visually inspected and artefacts including squid jumps were detected and rejected.

A single layer realistic head model was used in which the brain was divided into a number of grid points. The distance between adjacent points was 5mm. Lead-fields were calculated using FieldTrip software [30] and the co-registration of the MEG data and MRI was performed by using the affine transform whose matrices for head deviation and movement were available in the recorded MEG data.

Normalised output power of LCMV beamformer was used to identify the location of interference (equation (2)), and the output power of null-beamformer was used as a measure of neural activity (equation (4)). It is clear that in these two equations noise has been assumed to be white Gaussian, therefore the power has been simply normalised by the norm of the lead-fields.

The 10-minute continuous data were segmented using non-overlap rectangular windows with the length of 5 sec. The spectral spatial matrices were then calculated for each segment to be able to reconstruct the neural activity using the null-beamformer as in equation (4). No regularisation was applied to the spectral spatial matrix, and smoothing window for each frequency bin was ±2Hz [30].

### LCMV Beamformer and LCMV Null-beamformer

An LCMV beamformer is a linear filter *W* that minimises the power of all locations, but passes the signal originating from the location of interest [6]:
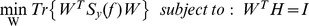
(1)where 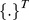 and 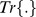 denote the transpose and trace operations, respectively. *H* is the lead-field at the location of interest and *I* is the identity matrix. Moreover, 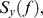 which is a positive definite matrix, is the spectral spatial matrix of the multichannel MEG data *y* calculated at frequency *f*. The spectral spatial matrix can be estimated as 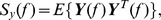 where 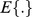 is the expectation operator and 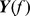 is the Fourier transform of the measurement *y* at frequency *f*.

The solution of *W* can be given using Lagrange multiplier method and normalised power at the location with lead field *H* is then given by [6]:
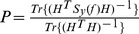
(2)


To spatially suppress the noise originating from the DBS device, one potential approach would be to place a null at the burr hole location whose lead-field is 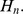 This null constraint is added to the beamforming formulation, equation (1), which results in the following problem formulation: .
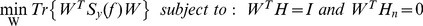
(3)


The closed form solution of the above problem is calculated using the Lagrange multiplier method and the normalised power of the source is then given by:
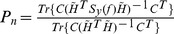
(4)where 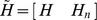 and 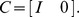 Note that equation (4) can be obtained from (3) in a same way that equation (2) is obtained from (1). Instead of using one grid point as the location of interference, it is possible to use a set of *q* grid points with associated lead-fields 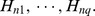 In this case we have 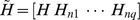 and 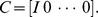

